# Optimization of sewage sampling for wastewater-based epidemiology through stochastic modeling

**DOI:** 10.1186/s44147-023-00180-1

**Published:** 2023-02-15

**Authors:** Max Martin, Paul Goethals, Kathryn Newhart, Emily Rhodes, Jason Vogel, Bradley Stevenson

**Affiliations:** 1 United States Corps of Cadets, United States Military Academy, West Point, New York, USA; 2 Department of Mathematical Sciences, United States Military Academy, West Point, New York, USA; 3 Department of Geography & Environmental Engineering, United States Military Academy, West Point, New York, USA; 4 School of Civil Engineering and Environmental Science, University of Oklahoma, Norman, Oklahoma, USA; 5 Microbiology and Plant Biology, University of Oklahoma, Norman, Oklahoma, USA

**Keywords:** Wastewater, Epidemiology, Monte Carlo simulation

## Abstract

The proliferation of the SARS-CoV-2 global pandemic has brought to attention the need for epidemiological tools that can detect diseases in specific geographical areas through non-contact means. Such methods may protect those potentially infected by facilitating early quarantine policies to prevent the spread of the disease. Sampling of municipal wastewater has been studied as a plausible solution to detect pathogen spread, even from asymptomatic patients. However, many challenges exist in wastewater-based epidemiology such as identifying a representative sample for a population, determining the appropriate sample size, and establishing the right time and place for samples. In this work, a new approach to address these questions is assessed using stochastic modeling to represent wastewater sampling given a particular community of interest. Using estimates for various process parameters, inferences on the population infected are generated with Monte Carlo simulation output. A case study at the University of Oklahoma is examined to calibrate and evaluate the model output. Finally, extensions are provided for more efficient wastewater sampling campaigns in the future. This research provides greater insight into the effects of viral load, the percentage of the population infected, and sampling time on mean SARS-CoV-2 concentration through simulation. In doing so, an earlier warning of infection for a given population may be obtained and aid in reducing the spread of viruses.

## Introduction

Public health officials continue to work toward a more efficient and representative way to identify and follow the spread of viral infections. With the sufficient mapping of an outbreak, preventative measures can be implemented, and lives may be saved. In recent years, nasal polymerase chain reaction (PCR) tests have been used to track the spread of the Ebola and SARS viruses [1]. While these tests are both accurate and reliable, populations can be restricted in terms of access to testing due to geographical boundaries, financial limits, or other societal factors. In recent years, wastewater-based epidemiology (WBE) has become a more popular method in the public health sector for tracking the prevalence of a virus in a geographic region anonymously and overcoming social factors that currently limit access to widespread testing by measuring the concentration of a chemical or biological constituent in municipal wastewater. Initially, WBE was used to determine illicit drug use [2, 3] but in recent years has expanded to include pharmaceuticals and personal care products, industrial chemicals, lifestyle markers, and, most recently, biomarkers like SARS-CoV-2 [4, 5]. Recently, the National Academies of Sciences has recommended the formation of a national wastewater-based infectious disease surveillance system to inform public health action [6]. Since it can be used to capture information on individuals who are potentially asymptomatic or otherwise not tested, the technique offers a unique perspective [7]. In the future, WBE of SARS-CoV-2 could provide public health officials with essential information on the spread of the virus to appropriately apply targeted health measures.

The first modern paper on WBE was written by Christian Daughton in 2001, which concerned the surveillance of illicit drug use [2]. However, this paper was primarily theoretical in nature, as it took 4 years for cocaine to be successfully extracted and quantified from wastewater. Since this initial investigation on illicit drug use, further studies have expanded their scope to include alcohol, tobacco, and other drug usage along with pathogens like Ebola and SARS [5]. Among the numerous WBE publications in the literature following Daughton’s research, there are two primary attributes for differentiation: the choice of sampling technique utilized by the researchers and computational processes selected for analysis. With emphasis on research published within the last ten years, these focus areas are further highlighted.

### Sampling techniques

There are currently three standard WBE sampling techniques: grab samples, time-weighted composite samples, and flow-weighted composite samples. Grab samples, which are taken any time of the day, capture the wastewater that is passing by the collection point at that given time. In contrast, time-weighted composite samples are taken at regular time intervals and averaged arithmetically, and flow-weighted composite samples are taken at regular flow intervals for a more realistic measure of total mass given diurnal fluctuations in flow and concentration.

Curtis et al. [8] analyzed the variability between grab samples and 24-h time weighted samples and determined that calculations to determine viral load between the two samples exaggerated their differences. The difference in these samples can be attributed to dilution and interferences with other constituents in municipal wastewater. *Graywater* comes from non-toilet appliances (e.g., shower, dishwasher, sink) and is a major dilutive factor in measuring biological concentrations in wastewater [9]. Thus, graywater volume must be accounted for in an experimental study to determine the true concentration of the WBE analyte in question.

Another cause of variation in WBE is sample location in relation to the sewage system. A recent study noted that samples taken from the influent of municipal wastewater treatment plants (WWTPs) can be heavily influenced by the accumulated dilatation at the end of the sewershed [10]. In Bibby and Peccia [10], five sampled WWTPs each served a population between 100,000 and 1,000,000 people. Due to this, the researchers found a wide variety of different pathogens in the wastewater. This illustrates one complication that may result from sampling a large population: when the total volume of wastewater increases due to the accompanying graywater contribution, the virus concentration is further reduced or diluted. A Hong Kong-based study compared viral loading at different locations within a sewershed: a hospital ward, a residential building, and a wastewater treatment plant [11]. Similar to previous literature, as the sample population got larger, the results became more variable and less trustworthy due to the pathogen becoming too diluted for the current testing processes.

In contrast, similar to this study, some researchers have examined smaller populations for WBE sampling. Bivins and Bibby (2021), Gibas et al. (2021), and Karthikeyan et al. (2021) performed studies of specific university campus buildings to compare with large-scale infection rates [12–14]. Moreover, Barrios et al. (2021) and Spurbeck et al. (2021) sampled small neighborhoods as a mechanism for designing health intervention policies at the community level [15, 16]. Oh et al. [17] outline the many benefits of conducting WBE at the scale of a neighborhood or small population. One predominant realization in all of these studies is the reduction in variability observed across the virus measurements.

Equally as important as sample location are the temporal characteristics of a sample. Evans et al. [18] identified temporal profiles of wastewater systems that vary largely from building to building. This is due to many factors, including, but not limited to: the number of people in the building, the building’s use, and the schedule that people use the building. These factors can greatly influence the amount of graywater that appears in a sample and may affect the calculated positive COVID-19 cases for a particular geographic region.

### Computational processes

Most WBE literature involve studies that utilize common analytics to explain their results. Hart and Halden [19] employed a computational analysis model from the U.S. Environmental Protection Agency to estimate sewage travel time, flow rates, and velocity. They then used cost metrics to estimate savings in testing a population for a virus. Salvatore et al. [20] document using techniques in principal component analysis specifically designed to analyze temporal data. The methods enabled one to establish regression models for predicting temporal changes in wastewater measurements.

There are limited previous research studies, however, that incorporate simulation and WBE data to predict the number of positive cases associated with a certain pathogen. Wang et al. [21] utilized Monte Carlo simulation to evaluate tobacco consumption in a population paired with WBE. Ahmed et al. [22] utilized the same simulation method to estimate the number of people who were infected with a virus, assuming various distributions for model attributes. Both studies by Wang et al. [21] and Ahmed et al. [22] supported looking at very broad populations for the purpose of evaluating detection in general.

The previous literature has not considered variations in a community population. Specifically, they did not focus on any sample populations less than a couple thousand residents. The long-term goal of WBE will be to tailor public health policies to specific areas, thus both controlling the disease while also minimizing collateral damage of public health policies. This work proposes a framework for analyzing different-sized populations and by conducting sensitivity analysis. We conjecture that the mean SARS-CoV-2 concentration will vary from community to community, simply based on the parameters defined for a specific sample population.

There are many challenges to implement WBE. Many factors can impact interpretation of the pathogen concentration data collected using WBE. First, depending on the location where the samples are taken, it can be difficult to estimate the population that is being sampled, especially in commercial districts. In addition, the concentration of SARS-CoV-2 copies in a sample may depend on a wide variety of considerations such as dispersion factors, infrastructure design, and population activity. To fully gain an understanding of these effects, it may require years of conducting experimental sampling procedures in tandem with community testing for COVID. For these reasons, there is an opportunity for simulating WBE efforts, a technique that can be further refined with greater future knowledge of sampling outcomes. If coupled with a real-world SARS-CoV-2 sampling study, methods in simulation may facilitate estimating fairly accurate infection rates prior to an outbreak.

In the sections that follow, a methodology for simulating a sampling process is described that seeks to produce a mean SARS-CoV-2 concentration for a given sample population. Finally, a real-world sampling case study is performed at the University of Oklahoma (OU) to compare with the simulation output and support validating various parameter settings. Research extensions are also described thereafter, so that valuable insights for improving WBE viral load detection may be obtained.

## Methods

A stochastic modeling approach is presented that models WBE processes for varying size populations. This involves designing a Monte Carlo simulation in ProModel (version 10.8.81), a discrete-event simulation software package, using distributions for various parameters described in this section. Prior to outlining the steps of our methodology, several relevant assumptions in modeling are described.

### Assumptions

While the intent is to reduce error as much as possible in simulating SARS-CoV-2 sampling for a given population, some assumptions must be made. In these cases, previous literature on the topic is investigated or known information regarding the population is sought to arrive at feasible and acceptable settings. The following paragraphs describe many of the parameters that must be considered in the development of a simulation approach.

Of particular interest is the average number of times an adult will defecate per day. Walter et al. [23] concluded that 98% of healthy individuals defecate between three times per day and three times per week. This study was confirmed by Mitsuhashi et al. [24] that also supported the “3 by 3” metric. A more specific study conducted by Bharucha et al. [25] on a group of women concluded that 80% of subjects without gastrointestinal issues defecated between 0.9 and 1.7 times per day. Other studies suggest that it depends on the age and gender of the population, as male populations less than 35 years old are found to have a greater stool frequency than other demographic areas. More active populations are reported to pass stools more frequently than sedentary groups [26]. This information provides, at best, a starting point when looking at a particular community of interest.

The proportion of the population that sheds a virus is also a parameter of interest. Szymczak et al. [27] concluded that only between 40-50% of COVID-positive people shed the virus in their feces. Cuicchi et al. [28] found that 46.5% of those with confirmed COVID-19 cases shed the virus in their feces. Young et al. [29] confirmed that 50% of confirmed COVID-19 cases shed SARS-CoV-2 genetic copies in their stool, but non-detectable amounts in their urine. The number of copies/L of SARS-CoV-2 in any one defecation must also be considered and can be highly variable [30]. Pan et al. (2020) reported levels between 10^5^ and 10^8^ copies/L, Zang et al. (2020) detailed levels between 10^8^ and 10^9^ copies/L, and Han et al. (2020) concluded that copy levels can reach up to 10^10^ copies/L [31–33].

Another parameter of interest is the size or volume of each defecation as this affects sampling efficiency and sewage throughput; for this parameter, several sources were referenced. First, Sender et al. [34] calculated that the average adult human has a defecation volume between 0.15 L and 0.25 L per day, assuming a once per day defecation schedule. However, Strid et al. [35] concluded that volume depends on population activity. For example, athletes tend to eat more food and thus defecate more frequently and of greater size. Sanjoaquin et al. [36] supports this same conclusion denoting that those who drink more water (like athletes) have larger defecations. Finally, a guide published by the Registered Nurses’ Association of Ontario suggests that “normal” defecations for an adult population are between 0.25 L and 0.50 L, the average of which is 0.375 L, still larger than Sender et al. [37] concluded.

There are also assumptions to consider regarding the sampling system, the infrastructure, and the flow composition. Many recent efforts utilized grab sampling for long term WBE studies [9, 18, 22, 38]. From real-world analysis, it takes approximately two minutes for a common autosampler to take a 100 mL sample. Moreover, the travel time in a wastewater pipe is often considered to be negligible with respect to mean SARS-CoV-2 concentration, even on the scale of a wastewater treatment plant [39]. Schussman and McLellan [39] also assumed there would be a moderate temperature prevalent in the wastewater pipe that would not have an impact on virus survival. Regarding the composition of the flow in the wastewater pipes, Oteng-Peprah et al. [40] estimated that 75% of the wastewater in standard household pipes is considered “graywater,” coming from other sources such as showers or baths, and laundry or sink water. This figure ranges between 50 and 80%, depending on the different water uses in residential facilities [41].

Finally, the concentration of SARS-CoV-2 in wastewater may be expressed in various ways depending on the sampling strategy. In particular, when performing grab sampling such as in this study, some researchers may assume the sample results represent the average concentration for an entire day [42–44]. To mitigate against variability in detecting pathogens, Bivins et al. (2021) and Augusto et al. (2022) recommend performing daily grab samples during the peak flow rate in any given community, usually between noon and 6 p.m. [12, 45].

### Modeling approach

**Fig. 1:**
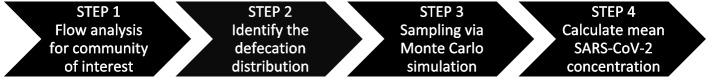
Modeling approach procedures

The goal of the simulation is to model the mean SARS-CoV-2 concentration for the community of interest. A first step is to collect and analyze flow data for a particular community of interest. This is necessary to establish a baseline for the environment as different-size populations will undoubtedly have varying levels of flow. Daily trends within a week may also exist—capturing representative data for each of the days in the week can facilitate providing a good representative picture of the flow rate. Next, utilizing some knowledge of the community, previous research, and the collected flow data, a distribution for defecations is defined for the simulation. After further identifying parameter settings tied to various assumptions, simulation trials are generated to establish the long-term behavior for the mean SARS-CoV-2 concentration. Figure 1 illustrates the procedural flow of these steps toward establishing our model.

#### Step 1: flow analysis

Production of graywater can vary greatly depending on geographic location, lifestyle, climate, sewage infrastructure, among other cultural factors. Graywater production varies between 20 L∙c/d in Gauteng, South Africa (province containing Johannesburg and Pretoria), and 151 L∙c/d in Muscat, Oman (the capital of Oman) [36]. Tuscon, Arizona, measured at the production level of 123 L∙c/d [36]. With such a wide variation, it is imperative that flow data be collected and analyzed prior to a simulation being designed. Two important factors that can be determined from the flow data are (i) a general knowledge of the flow rate of the community of interest which leads to measurements that will be used to compute the daily mean SARS-CoV-2 concentration and (ii) information regarding the percentage of graywater in the wastewater pipe. Both factors will greatly affect the accuracy of one’s calculations.

#### Step 2: defecation distribution identification

The identification of the distribution is derived from two main factors: knowledge of the given population and the collected flow data. As discussed previously, the age, gender, and activity of the population will influence the size and amounts of defecation in any given time interval. Examining the collected flow data may also provide insights into explainable trends and peaks such as the times when population density is greatest or completely inactive. Using these two factors, the defecation distribution can then be identified for the further use of simulation.

#### Step 3: Monte Carlo simulation sampling

The total number of individuals in a community of interest is identified. Given information from step 2 estimating when these individuals will defecate throughout the day, one or more cumulative density functions (CDFs) may be established for a 24-h period. The total number in the population is further broken down into three different groups: COVID-negative, COVID-positive shedding, and COVID-positive non-shedding. As mentioned previously, only a percentage of the population of interest will shed the virus in the local wastewater. The chosen defecation distribution for a population based upon weekly flow data and knowledge of the community then influences the number of COVID-19 positive defecations detected in a sampling operation.

In terms of the software, ProModel, entities are created to represent each defecation based upon the size of the population. Entity attributes are utilized to delineate between COVID-negative, COVID-positive shedding, and COVID-positive non-shedding feces in the right proportions. They are further held in a group queue and released from this module according to the distribution of interest. Upon departing the group queue, the entities enter another brief queue representing the sampling operation. The time in this latter queue is equivalent to the time it takes sampling equipment to collect a sample. On a given day, specimens that contain SARS-CoV-2 particles may then be detected when a sample is taken. Once entities depart the second queue, they are released from the simulated process until a new distribution is generated.

#### Step 4: SARS-CoV-2 concentration computations

The previous paragraphs explain the numerous assumptions and parameters that must be defined to support an accurate Monte Carlo simulation for wastewater-based epidemiology. These factors ultimately culminate in the calculation of the concentration of copies of SARS-CoV-2 virus per liter of wastewater, as shown at Eq. 1:

1 $$C=\frac{\textrm{copies}}{\textrm{L}}=\frac{\textrm{total}\#\textrm{of}\ \textrm{copies}}{\textrm{total}\ \textrm{wastewater}\ \textrm{Vol}.}=\frac{c_{+}{kV}_d}{Qt_s}$$

where *C* is the concentration of copies of SARS-CoV-2/L, derived from the total number of SARS-CoV-2 copies in the wastewater divided by the volume of the wastewater in the sewer. In the numerator, *c*_+_ represents the number of COVID-19 positive defecations at that time, *k* is the number of SARS-CoV-2 copies/L in a defecation, and *V*_d_ represents the volume of a defecation per person. In the denominator, *Q* represents the community flow rate in liters per second at the moment that the sample is being collected, and *t*_s_ represents the time it takes for a sample to be taken in seconds. Using these variables, the concentration *C* in copies of SARS-CoV-2/L can be calculated.

To calculate Eq. 1, the number of COVID-positive defecations will be taken from the output of the Monte Carlo simulation. The other two terms in the numerator are identified based upon knowledge of the community of interest and the flow analysis results. The denominator determines how much wastewater is flowing by the sampling location as the sample is taken. Finally, similar to some previous research efforts, the simulation will calculate a SARS-CoV-2 concentration in copies/L at a specified time of the day, and this concentration will be assumed to be the average concentration throughout the day. Daily concentrations will then be used to identify a running average concentration observed over a longer period of time.

## Results and discussion

### Case study: a community of interest

Data utilized for this project was part of a larger unpublished data set from monitoring for SARS-CoV-2 in wastewater from residence halls on the campus of the University of Oklahoma during the fall semester of 2020 and the spring semester of 2021. The community of interest for this particular study consisted of approximately 325 students, which were both male and female, generally between the ages of 18 and 22 years old, and many that were college athletes. In accordance with the procedures outlined in the “Modeling approach” section, the first step is to analyze the flow for the community of interest. As mentioned previously, the flow may be highly variable across different-sized populations with diverse supporting infrastructure. Using a flow sensor installed in a sewage pipe from a manhole south of a residence hall, the flow rate was collected each minute for a 6-month period between December 2020 and May 2021 using refrigerated Avalanche autosamplers from Teledyne Isco of Lincoln, Nebraska. Discarding a small number of days when the flow sensor malfunctioned, the average daily flow rate was recorded for each day of the week (see Appendix). The manhole location was selected to represent the combined flow from all the residents that utilized the facility.

**Fig. 2:**
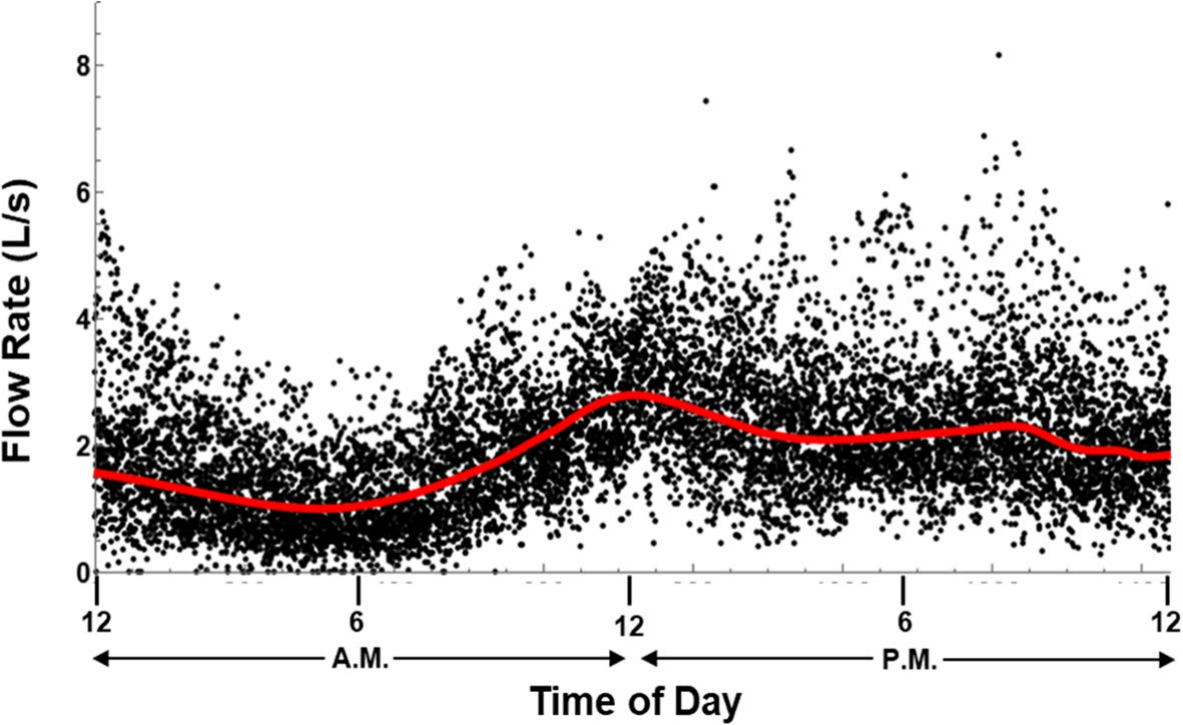
Merged flow data with mean trend line (OU community of interest)

Identifying the defecation distribution, the second step of the modeling approach, requires considering both characteristics of the community of interest and observing the flow data. The residents are students who exhibit high activity in the morning, afternoon, and evening hours with requirements to attend classes and participate in daily athletic practices. Depending on the day of the week, the population may also exhibit high activity in the late-night hours. Each day of the week depicts some trend whereby some outliers may be present. When all the flow data is merged into one plot and a higher-order trend line is used to depict the average at each minute of the day (Fig. 2), a bimodal pattern is observed with peaks at the midday and evening hours. In addition, there are instances where no flow is observed or where it is at a minimum, primarily between the hours of midnight and 6 a.m.

**Fig. 3:**
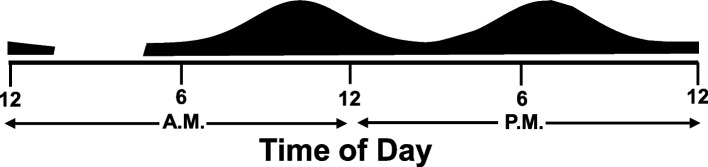
Defecation distribution alignment with time of day

It is important to note that the data depicted in Fig. 2 includes graywater which, as previously indicated, is assumed to be 75% of the total flow. Another significant consideration is the fact that this population is primarily young and athletic; as discussed in the “Assumptions” section, athletes are found to defecate more than an average person. Given this information and the supporting data, it is assumed that this population defecates, on average, twice a day. Furthermore, with the peak flow occurring when students are likely returning from morning classes and then returning from afternoon activities and dinner, the distributions for each defecation are assumed to be based upon a normal distribution with means at 10:30 a.m. and 7:30 p.m., respectively. To account for the spread of the data observed, a 2-h standard deviation is used with each distribution. Figure 3 depicts the approximate defecation distributions and their alignment with the time of day. The six-sigma limits for a normal distribution account for more than 99% of the observational data. Given a 2-h standard deviation, the distribution will span a total of 12 h, enabling one to achieve a bimodal pattern for the midday and evening hours.

Upon establishing a program to generate the defecation distributions in ProModel, the third step in the modeling approach calls for the development of a Monte Carlo simulation to perform a sampling process. To complete this step, several parameter values outlined in the “Assumptions” section are identified.

First, like the actual sampling process conducted at the residence hall, a “grab sample” is performed at noon each simulated day. It is important to note that this process accounts for times when SARS-CoV-2 is detected or not detected, but it is not specifically generating random samples representative of false negatives (not detecting virus when it is present) or false positives (detecting the virus when it is not present). A “sampling location” is established in the simulation similar to a wastewater pipe, whereby once a defecation occurs, it enters a group queue for a two-minute period before flowing out of the system. The 2-min period is designed to replicate the time taken for a real-world autosampler to physically sample a specimen. The number of defecations observed at the sampling location is recorded and used in future calculations.

Secondly, each defecation volume is established at 0.375 L, in accordance with the research discussed previously on athletes and average adults [34, 37]. In addition, based upon estimations from September 2020 to May 2021 and the actual COVID copy counts discovered via sampling, a % COVID-positive rate is established for the simulated population. Moreover, given the community of 325 students, one half of all COVID-positive individuals will shed the virus in their feces, in accordance with previous research.

Although there are likely effects such as dispersion that can be considered, for the purpose of this initial study, instantaneous sampling is performed. That is, the duration of travel between the time the defecation occurs and when it is sampled is zero. This assumption aligns with the recent research of Schussman and McLellan [39], whereby mean SARS-CoV-2 concentrations change very little from source to sample, even on the scale of a wastewater treatment plant. The size and scale of this particular community of interest do not necessarily warrant considering effects such as viral decay or mortality during the time frames of water transport and sample detection. In contrast, if larger systems or processes make up a community of interest, additional evaluation may be required.

The fourth and final step in the modeling approach is to calculate the mean concentration of SARS-CoV-2 in copies/L. Given our case study scenario with a population of 325 students, a proportion of this population is COVID-positive and sheds the virus in their feces. A grab sample is taken at noon on a given day of the week during a 120-second duration of time. With a suspected viral load and the community flow rate at that specific time, the mean SARS-CoV-2 concentration is calculated. For instance, based upon the randomly generated defecation distribution for a population suspected to have a 15% COVID-positive rate where the simulation sample finds two COVID-positive defecations at noon on a Monday, the viral load of a feces for a person (p) is assumed to be 10^8^ copies/L, and the community flow rate at that time is 2.149 liters per second, the mean concentration for that day is:

$$C=\frac{\left(2\;\textrm{p}\right)\left({10}^8\kern0.24em \textrm{copies}/\textrm{L}\right)\left(0.375\kern0.24em \textrm{L}/\textrm{p}\right)}{\left(2.149\kern0.24em \textrm{L}/\textrm{s}\right)\left(120\kern0.24em \textrm{s}\right)}=290,853\kern0.24em \textrm{copies}/\textrm{L}$$

**Fig. 4:**
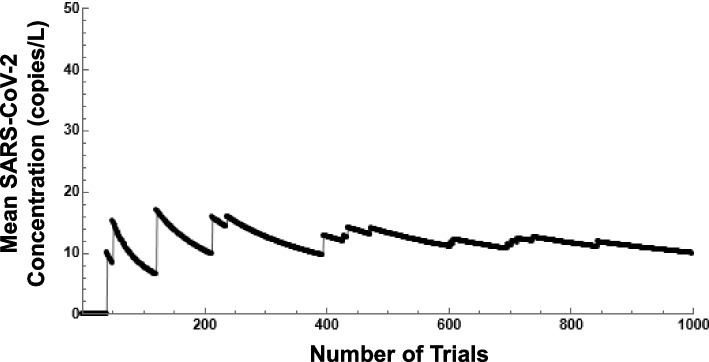
Simulation output, running mean SARS-CoV-2 concentration (L/s), 1000 trials

Prior to performing an analysis of the sensitivity related to solutions generated by the Monte Carlo simulation, several experiments are conducted on the long-range behavior of the mean SARS-CoV-2 concentration. Under various settings, up to 1000 trials (each trial representing 1 day) of the simulation are generated with a calculated running average of the mean concentration. These settings include varying the population infected (5–25% infected), the viral load (10^6^–10^10^ copies/L), and the time that the sample is taken (8 a.m.–11 p.m.). In each instance, the mean appears to converge at some value after approximately 500 trials. Figure 4 illustrates a convergence to roughly 10 copies/L when the population infected is 5%, the viral load is 10^6^ copies/L, and a noon sample time is performed.

Based upon the results of these initial experiments, a total of 500 trials was chosen for all experimental runs in this study. Given multiple parameters of interest, an analysis of the sensitivity in the results was performed.

### Sensitivity analysis

Each parameter in the calculation of the mean SARS-CoV-2 concentration has some variability associated with it. Variation can occur in the viral load for each feces, as previous research outlined in the “Assumptions” section denotes intensities in the range from 10^6^ to 10^10^ copies/L. The time of the day that a sample is taken can also be a significant factor depending on the distribution chosen for individual defecations. For instance, given the defecation distributions that we identified for our community of interest, samples at 10 a.m., noon, and 11 p.m. correspond to 0.25, 0.75, and 1.75 standard deviations for the distributions, respectively. The smaller the standard deviation, the greater likelihood that COVID-positive feces may be discovered in our samples. The Monte Carlo simulation also requires some estimation of the number of COVID-positive individuals in the population. Sampling in highly populated communities or areas with larger catchments may influence the detection rate for SARS-CoV-2 [46, 47].

**Fig. 5:**
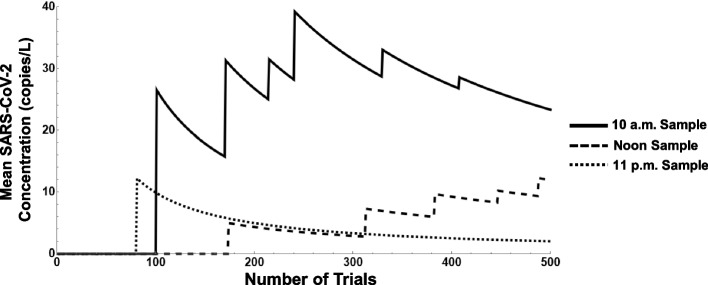
Simulation output, rolling mean SARS-CoV-2 concentration (L/s) when population infection is 5%, viral load is 10^6^, and grab sample time varied

**Fig. 6:**
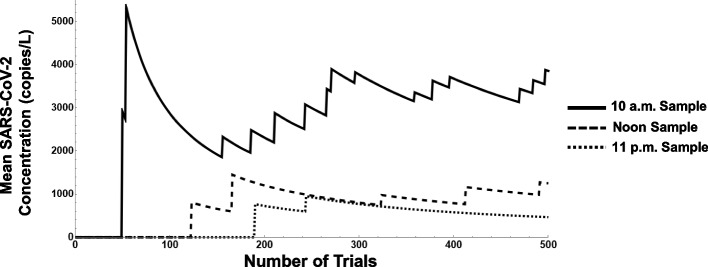
Simulation output, rolling mean SARS-CoV-2 concentration (L/s) when population infection is 10%, viral load is 10^8^, and grab sample time varied

**Fig. 7:**
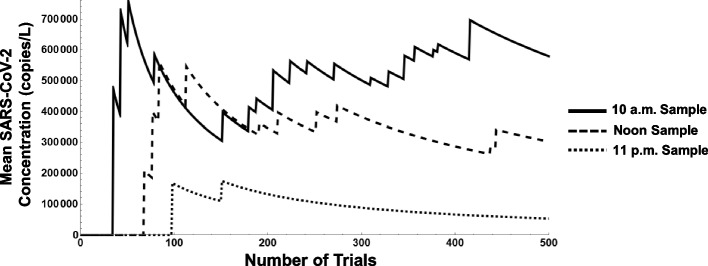
Simulation output, rolling mean SARS-CoV-2 concentration (L/s) when population infected is 15%, viral load is 10^10^, and grab sample times are varied

To analyze the effects of viral load, sampling time, and COVID-positive rates in our scenario, multiple experiments were performed. Figures 5, 6, and 7 depict the observed results of varying these parameters on the mean SARS-CoV-2 concentration. In particular, Fig. 5 displays three different experiments where 5% of the population is deemed COVID-positive and a viral load of 10^6^ copies/L is assumed. Each experiment involves performing grab samples at different times of the day repeated over a duration of 500 trials, the first using a sampling time of 10 a.m., the second at noon, and the third at 11 p.m. The effect of the parameter settings and the sampling time on the simulated mean SARS-CoV-2 concentration is observed. After 500 trials, mean concentrations of approximately 2, 10, and 25 copies/L result from the 11 p.m., noon, and 10 a.m. samples, respectively.

In Fig. 6, three similar experiments are performed, but with 10% of the population deemed COVID-positive and setting the viral load at 10^8^ copies/L. After 500 trials, we observe mean concentrations of approximately 750, 1500, and 4000 copies/L for the 11 p.m., noon, and 10 a.m. samples, respectively.

And in Fig. 7, the results are examined with 15% of the population deemed COVID-positive and a viral load setting of 10^10^ copies/L. After 500 trials, we observe mean concentrations of approximately 75,000, 300,000, and 600,000 copies/L for the 11 p.m., noon, and 10 a.m. samples, respectively. The breadth of the outcomes in mean SARS-CoV-2 concentration account for the wide range of inputs in the COVID-positive infection rates, viral load, and sampling times.

To identify the specific effects of the various parameters on the mean SARS-CoV-2 concentration, additional observations are made. In a follow-on experiment, the noon sample is selected and the simulation is run with 10% of the population deemed COVID-positive and a viral load setting of 10^8^ copies/L. After 500 days, a mean concentration of 125,290 copies/L is obtained. When this result is compared to Figs. 6 and 7 for the noon sample, we observe a 100-fold increase in viral load corresponds to a roughly 100-fold increase in mean SARS-CoV-2 concentration while a roughly ½-fold decrease in the percentage of population infected corresponds to a ½-fold decrease in concentration. In contrast, when viral load and the percent of the population infected are fixed such as in Figs. 5, 6, and 7, a shift of just 2 h in the simulated sample time results in a decrease of more than one-half the concentration in copies/L. The different units of measurement among the parameters, however, creates difficulty in directly comparing their individual “sensitivities.”

While it is apparent that each of the measures has some effect on the concentration, the greatest change in the overall mean is undoubtedly attributed to the scale, range, and variability of the viral load parameter. This can be directly observed by adjusting the viral load from 10^6^ to 10^10^ copies/L in Eq. (1), whereby a ten thousand-fold increase results in the mean SARS-CoV-2 concentration. The overwhelming effects of viral load on SARS-CoV-2 concentration are further substantiated by other researchers performing real world studies [48–50].

### Comparison with local measurements

**Fig. 8:**
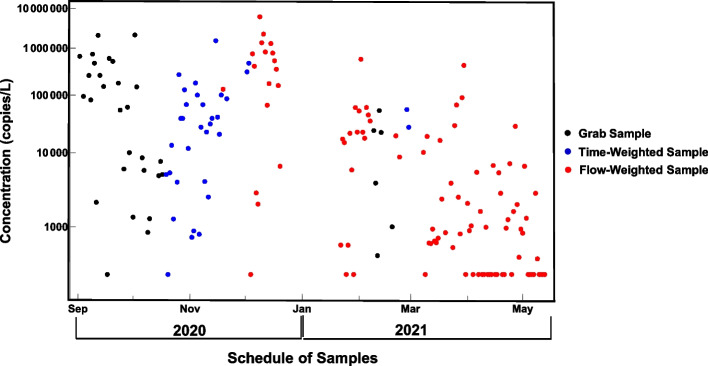
Actual SARS-CoV-2 concentration for sample population using different sample collection methods

For a 9-month period between September 2020 and May 2021, in addition to analyzing the flow rate for the community of interest, a sampling campaign was performed to monitor the actual mean SARS-CoV-2 concentration. During this period, 34 grab samples, 33 time-weighted samples, and 100 flow-weighted samples were collected on different days from the dormitory sewer. Aside from the composite samples, which were drawn over a 24-h period, the samples were collected at approximately noon on each day. After collection, samples were immediately transported to the analytical laboratory on the campus of the University of Oklahoma and analyzed for the N1 SARS-CoV-2 marker using a kit-less analytical method described in Kuhn et al. [38]. The sampling results are displayed in Fig. 8. Of note, the mean SARS-CoV-2 concentration was found to be 247,000 copies/L for the grab samples, 109,000 copies/L for the time-weighed samples, and 165,000 copies/L for the flow-weighted samples. These figures would serve as a baseline for evaluating the Monte Carlo simulation output.

From Figs. 5, 6, and 7, it may be possible to infer a confidence interval for the case rate of the community of interest, especially if greater information on the viral load is available. The different scales observed on the *y*-axis are a direct result of the different viral loads; given our parameters, this factor will produce results that may vary as much as 10^4^ in magnitude. For instance, if the population infection rate is assumed to be 5%, as shown in Fig. 5, but the viral load is established at 10^8^ copies/L, the graph will look very much the same but on a scale one hundred times larger in magnitude. For our scenario, if we assume the viral concentration of one fecal event is slightly above average at 10^10^ copies/L, we can expect from this analysis that the population infection rate is likely within a range of 8-13% during this period (a mean SARS-CoV-2 concentration close to 300,000 copies/L is observed in Fig. 7, whereas the actual sampling process resulted in noon grab samples with mean concentrations close to 250,000 copies/L).

However, there exist several factors that may suggest the simulation is slightly overestimating mean concentration. First, the simulation assumes that the 325 students are always using the bathroom facilities at the residence hall during any given day, when in fact, they may use any campus facility. Secondly, the simulation does not account for holiday breaks or periods of time when the facilities may be completely empty. Finally, the simulation assumes that all students in the residence hall have slightly above average defecation size and frequency. In reality, it may depend on the type of student activity whereby more energetic individuals truly only produce these results. With greater knowledge in future research of these subject areas, higher precision may be possible with simulation.

### Future applications

A complementary goal of this research was to create a generic, stochastic modeling approach that could be applied to different populations. To extend this work to less homogeneous populations, the bimodal distribution assumed in this work may be more skewed: with the variance of the evening peak greater than the morning peak (as is commonly observed in flow rates of larger sewersheds). The rate and mass of each defecation event could also impact the assumed distribution. Observed differences in flow, defecation frequency, and SAR-CoV-2 load per event correspond to greater variation in lifestyle of the population; which could be impacted by gender, diet, BMI, exercise habits, and genetics [36]. To extend this work to a heterogeneous population when the behaviors and demographics can be assumed of the sub-populations, the bimodal distribution could be substituted for a multivariate probability distribution. To extend this work to larger communities, identifying representative sampling locations (i.e., interceptors where the sample is well mixed) where the flow is monitored would allow a detailed accounting of dilution from inflow and infiltration (I&I) and non-residential discharges [47, 51].

Factors relating to mechanical dispersion within the sewer collection system need to be considered as well, especially for larger communities of interest. In this case study, the effects of dispersion were deemed negligible, given that the pipe supporting the residence hall was small and a small population was studied. For extremely large populations, sampling can occur at an interceptor where the wastewater is well mixed. However, when sewershed sampling occurs in rural areas or targeting smaller sewersheds lacking mixing, dispersion should be explored as an experimental factor.

Additionally, the time of sampling requires greater attention. In a practical application of WBE, samples will only be taken once or twice a day due to the financial and time cost of taking samples. Therefore, it is important that the samples accurately represent the infection rate of the community of interest. In this study, we analyzed three different sampling times. Future studies may consider factors such as the different sample types and alternative equipment for optimizing sampling collection strategies both temporally and spatially. Greater analysis in sampling times is needed, as well as how the results from those samples contrast with the actual case count of the community of interest. With accurate sampling times and results, public health officials can better control the spread of disease in the community.

## Conclusions

This work seeks to take an initial step forward in using stochastic modeling to study WBE. A general framework is proposed that can be modified for any size community of interest. It begins with a study of the flow data which, when combined with knowledge of the population, leads to identifying a defecation distribution for the individuals. A Monte Carlo simulation is then produced to model the sampling process based upon this distribution and various parameters. Finally, as a result of the flow characteristics, the viral load, and the repetitive nature of the simulation, a mean SARS-CoV-2 concentration is obtained.

In the “Sensitivity analysis” section, a specific community of interest was observed whereby the effect of three parameters on the mean SARS-CoV-2 concentration was studied. An experimental investigation of the parameters, namely viral load, the percentage of the population infected, and sampling time, led to several research findings. First, the breadth of variability in the mean SARS-CoV-2 concentration when altering various parameters is easily observed for a particular population. This may provide a greater future awareness of the changes observed across different populations, especially since communities of interest have unique characteristics in terms of demographics, graywater content, and infrastructure. Second, among the three parameters, viral load exhibited the most influence on the mean SARS-CoV-2 concentration, due to its scale, range, and variability. While the percentage of the population infected and the choice of sampling time also showed significant influence, a comparison of the two parameters offered inconclusive results. Third, the sensitivity analysis may enable one to make inferences on the degree to which a population is infected when quantities such as viral load are known or assumed. Greater knowledge of highly influential parameters such as viral load may certainly lead to increased precision in estimating infection rates, a typical goal of wastewater-based epidemiology studies.

In conclusion, the methods and findings for this research work can be applied in various ways. The proposed analytical framework can be extended to larger communities when factors such as those described in the “Future applications” section are examined. In addition, approaches in simulation offer an ability to perform “what if” analysis and explore different scenarios without real-world experimentation. Future stochastic modeling efforts with WBE studies may provide supplementary information that further optimizes viral collection and detection processes. The results for this study can also be used to impart awareness of the degree or significance to which factors may affect sampling outcomes. Finally, with greater future knowledge of subject areas such as human factors and infrastructure effects in wastewater epidemiology studies, approaches in simulation may gain added precision and accuracy in their formulation. In turn, public health officials may achieve better insights and then gain efficiencies in shaping public health policy.

## Figures and Tables

**Fig. 9:**
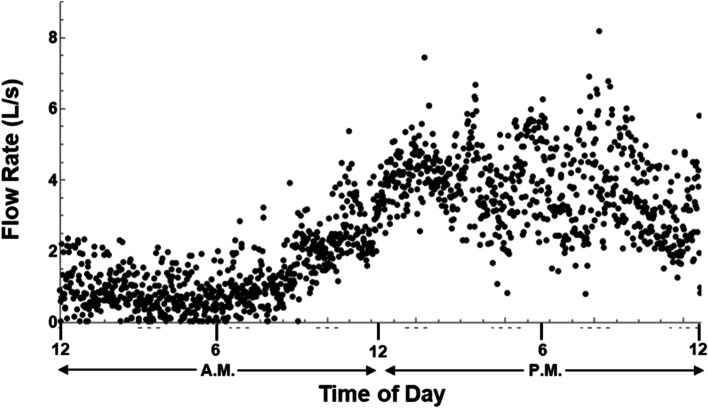
Monday average readings for daily flow

**Fig. 10:**
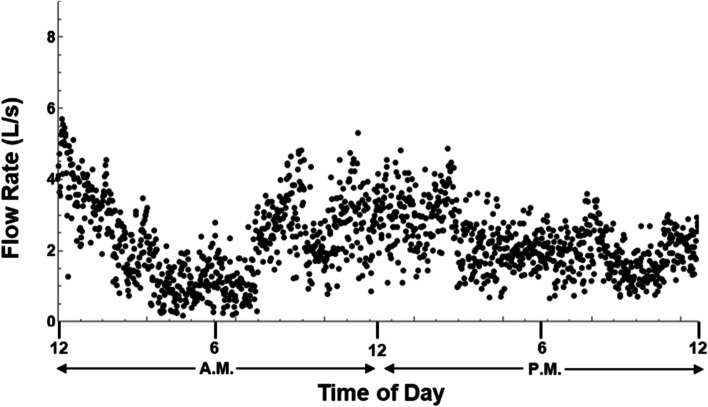
Tuesday average readings for daily flow

**Fig. 11:**
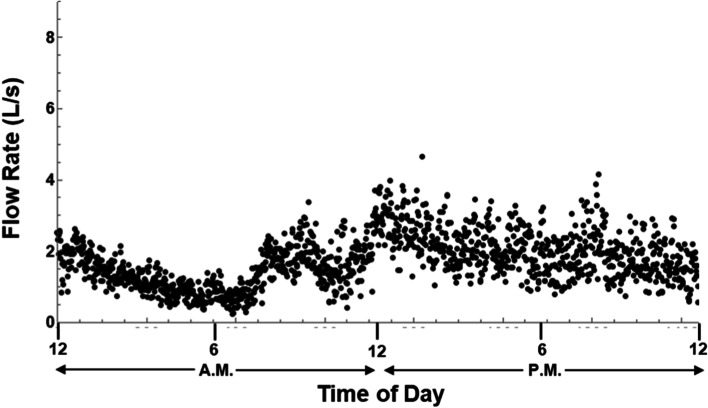
Wednesday average readings for daily flow

**Fig. 12:**
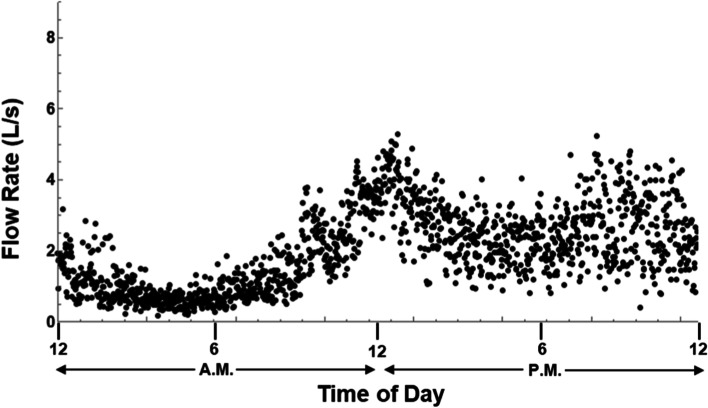
Thursday average readings for daily flow

**Fig. 13:**
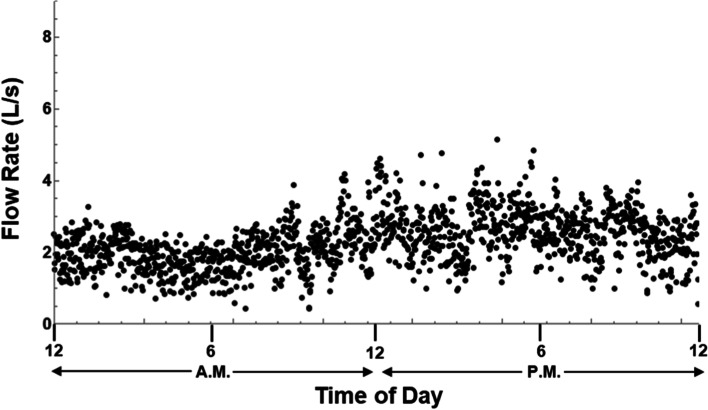
Friday average readings for daily flow

**Fig. 14:**
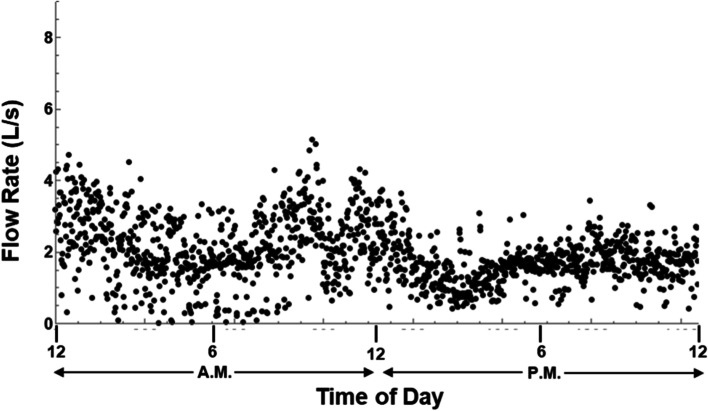
Saturday average readings for daily flow

**Fig. 15:**
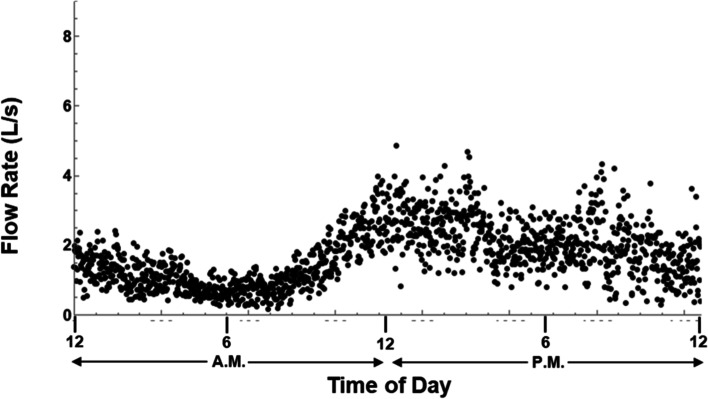
Sunday average readings for daily flow

## Data Availability

All data generated or analyzed during this study are included in the article (and in its supplementary materials).

## Appendix

The daily flow for a particular residence hall at the University of Oklahoma is analyzed over a six-month period, to provide a baseline for future measurements. Shown in the following figures are the average readings for each day of the week during the 6-month period:

Figures 9, 10, 11, 12, 13, 14 and 15

## Abbreviations

CDF: Cumulative density function
COVID: Coronavirus disease
I&I: Inflow and infiltration
OU: University of Oklahoma
PCR: Polymerase chain reaction
SARS: Severe acute respiratory syndrome
SARS-CoV-2: Severe acute respiratory syndrome-related coronavirus 2
WBE: Wastewater-based epidemiology
WWTP: Wastewater treatment plant
